# ﻿Review of the genus *Ectinogramma* Thomson, 1864 (Coleoptera, Cerambycidae, Lamiinae, Agapanthiini)

**DOI:** 10.3897/zookeys.1244.148097

**Published:** 2025-07-11

**Authors:** Gui-Qiang Huang, Yao-Lan Li, Hai-Jun Lu, Dong-Shuo Liu

**Affiliations:** 1 School of Biological Science and Technology, Liupanshui Normal University, Liupanshui 553004, Guizhou, China Liupanshui Normal University Liupanshui China; 2 502, Unit 1, Building 3, Xinhua Lian Lijing Sub-district, No.28 Shilibao Beili, Chaoyang District, Beijing, China Unaffiliated Beijing China

**Keywords:** Gender definition, new combination, new faunistic records, redescription, synonymy, taxonomy

## Abstract

A taxonomic review and redescription of the genus *Ectinogramma* Thomson, 1864 are presented. *Spinogramma* Breuning, 1947 is proposed as a junior synonym of *Ectinogramma* Thomson, 1864, and *Spinogrammaruficollis* Breuning, 1959 as a junior synonym of *Ectinogrammaisosceloides* Thomson, 1864. *Ectinogrammaochreovittata* (Breuning, 1947), **comb. nov.** is proposed as a new combination. The genders of the holotype specimens of *E.isosceloides*, *E.collare* Pascoe, 1866 and *S.ruficollis* are determined. Also, a male of *E.isosceloides* is described and illustrated for the first time.

## ﻿Introduction

Thomson (1864) established the genus *Ectinogramma* for *Ectinogrammaisosceloides* Thomson, 1864 from Malaysia (no further data), and thereafter, Pascoe (1866) described *E.collare*, from Penang, Malaysia. Subsequently, Breuning (1961) synonymized *E.collare* and *E.isosceloides*. Therefore, *Ectinogramma* currently consists of a single species, *E.isosceloides*, from Southeast Asia (Tavakilian and Chevillotte 2024). Breuning (1947) proposed the genus *Spinogramma* for *S.ochreovittata* Breuning, 1947, from Sumatra, Indonesia, and Breuning (1959) described *S.ruficollis*, from Kuala Lumpur, Malaysia. Therefore, *Spinogramma* is currently composed of two species from Southeast Asia (Tavakilian and Chevillotte 2024).

Our review of both genera, *Ectinogramma* and *Spinogramma*, suggests *Spinogramma* as a junior synonym of *Ectinogramma*. Moreover, the gender of the holotype specimens of *E.isosceloides*, *E.collare* and *S.ruficollis* was not reported in the original descriptions, and herein we determined it.

## ﻿Material and methods

The specimens examined are deposited in the following institutional and private collections:

**CDJH** Collection Daniel J. Heffern, Houston, Texas, United States;

**CSG** Collection Andre Skale, Gera, Germany;

**CST** Collection Sergi Trócoli, Barcelona, Spain;

**CWW** Collection Andreas Weigel, Wernburg, Germany;

**LPSNU** School of Biological Science and Technology, Liupanshui Normal University, Liupanshui, Guizhou, China;

**MNHN**Muséum national d’Histoire naturelle, Paris, France;

**NHRS**Naturhistoriska Riksmuseet, Stockholm, Sweden;

**RBINS**Royal Belgian Institute of Natural Sciences, Brussels, Belgium.

The methods of photography (Fig. 2B) followed Huang et al. (2020). The copyrights of other photographs were added to the legend of the corresponding figures.

**Figure 1.**
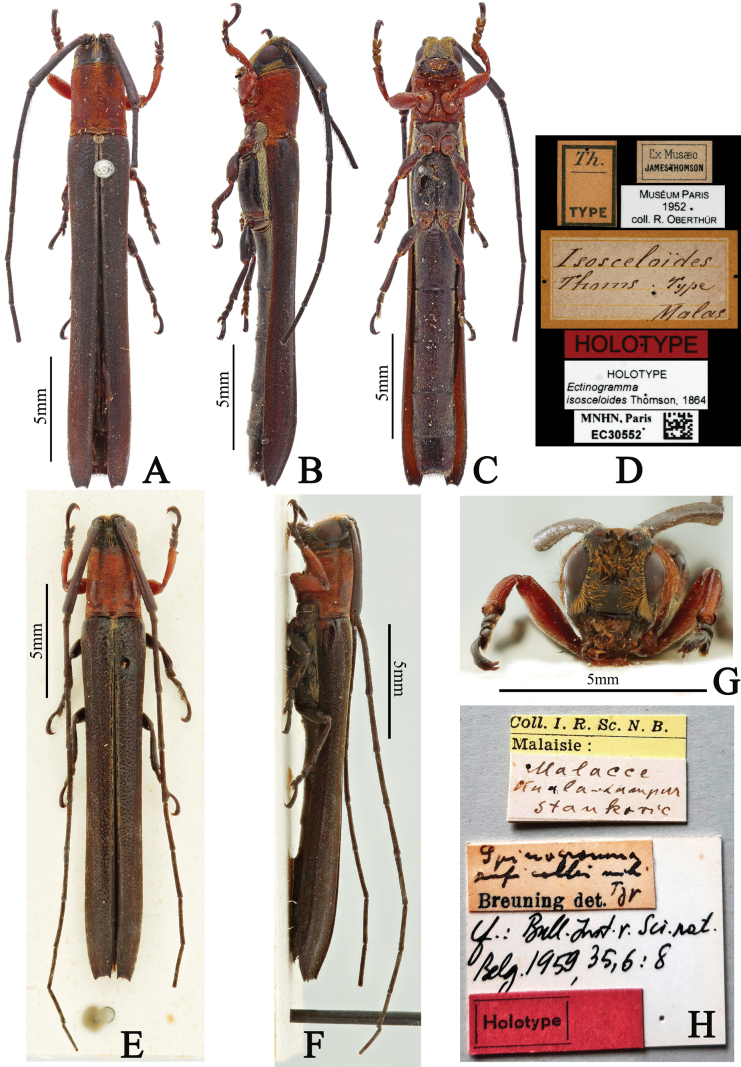
**A–D.***Ectinogrammaisosceloides*, holotype, female; **A.** Dorsal habitus; **B.** Lateral habitus; **C.** Ventral habitus; **D.** Labels (photographs **A–D** taken by Christophe Rivier); **E–H.***Spinogrammaruficollis*, holotype, female; **E.** Dorsal habitus; **F.** Lateral habitus; **G.** Frontal habitus; **H.** Labels (photographs **E–H** taken by Berdien Daniels).

**Figure 2.**
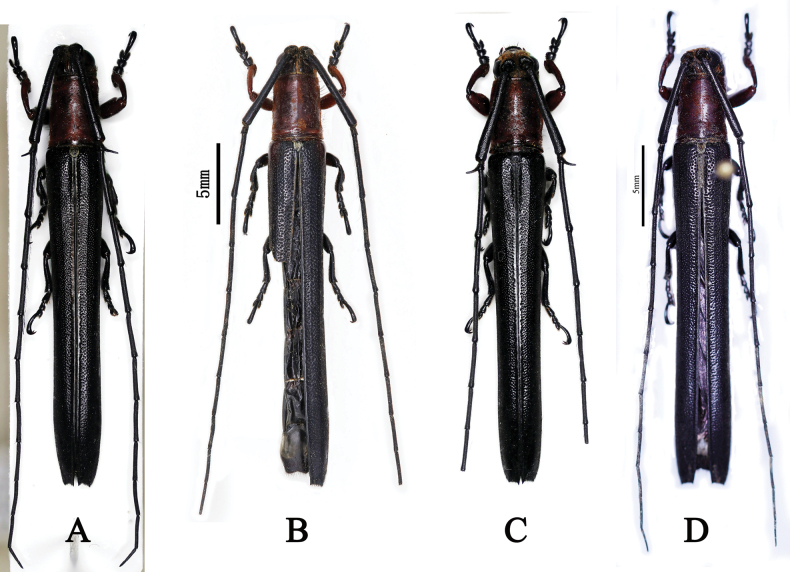
**A–D.***Ectinogrammaisosceloides*, dorsal habitus; **A.** Male from Sabah, Borneo, Malaysia (photograph taken by Andre Skale); **B.** Female from Sabah, Borneo, Malaysia (photograph taken by Yao-Lan Li); **C.** Male from Kalimantan, Indonesia (photograph taken by Andre Skale); **D.** Female from Kalimantan, Indonesia (photograph taken by Sergi Trócoli).

**Figure 3.**
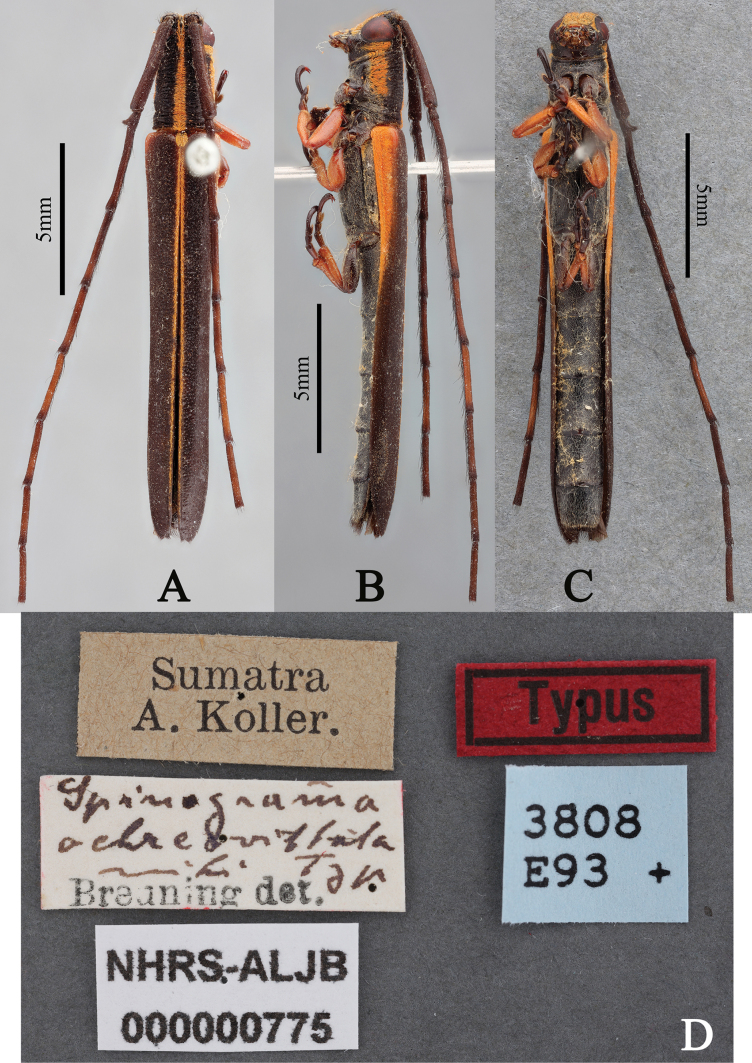
**A–D.***Spinogrammaochreovittata*, holotype; **A.** Dorsal habitus; **B.** Lateral habitus; **C.** Ventral habitus; **D.** Labels (photographs **A–D** taken by María Alejandra Álvarez Covelli).

The overall dimensions of the holotypes, i.e., body length and humeral width of elytra of *Ectinogrammaisosceloides* Thomson, 1864 (Fig. 1A), *Spinogrammaruficollis* Breuning, 1959 (Fig. 1E), *Spinogrammaochreovittata* Breuning, 1947 (Fig. 3A) and a female specimen of *E.isosceloides* (Fig. 2D) were obtained based on the scale bars contained in the photographs. The dimensions of a female specimen of *E.isosceloides* (Fig. 2B) were measured with a ruler. Other dimensional data of four specimens [i.e., a male (Fig. 2A) and a female in CWW, a male (Fig. 2C) in CSG, and a female in CST] of *E.isosceloides* were respectively provided by Andreas Weigel, Andre Skale and Sergi Trócoli. Other dimensional data were either provided by the following collaborators or calculated from their data as follows: (1) body length of a male specimen of *E.isosceloides* in CWW was provided by Andreas Weigel; (2) humeral width of elytra was calculated based on the specimen photograph provided by Andre Skale; (3) body lengths of two males and five females of *E.isosceloides* in CDJH were provided by Daniel J. Heffern; and (4) humeral width of elytra of the above seven specimens were calculated based on the specimen photographs provided by Daniel J. Heffern.

All photographs and figures were produced using Photoshop CS5 software.

## ﻿Taxonomy

### ﻿ Ectinogramma

Taxon classificationAnimaliaColeopteraCerambycidae

Thomson, 1864

00752FE6-419D-5A7B-B2D3-428306EDF780

Ectinogramma Thomson, 1864: 96; Pascoe 1866; Lacordaire 1872: 692, 700; Gemminger and Harold 1873: 3131; Aurivillius 1923: 359; Breuning 1961: 202 (catalogue). Type species: Ectinogrammaisosceloides Thomson, 1864.
Spinogramma Breuning, 1947: 61; Breuning 1961: 202 (catalogue). Type species: Spinogrammaochreovittata Breuning, 1947, syn. nov.

#### Remarks.

The morphological comparison of holotypes of *Ectinogrammaisosceloides* Thomson, (Fig. 1A–C) and *Spinogrammaochreovittata* Breuning (Fig. 3A–C) reveals that both species belong to the same genus, *Ectinogramma*, based on shared morphological characters. Therefore, *Spinogramma* Breuning is determined to be a junior synonym of *Ectinogramma* Thomson. Although detailed descriptions of both *Spinogramma* and *Ectinogramma* are provided by Breuning and Thomson, respectively, the description of *Ectinogramma* is enhanced below by examination of both types and additional examined material.

#### Redescription.

Body slender and elongate (body length / humeral width of elytra: 4.8–8.2) based on 16 specimens of *E.isosceloides* and one specimen of *E.ochreovittata*. Eyes finely faceted, lower eye lobe distinctly longer than gena (Figs 1B, F, G, 3B). Antennal tubercles distinctly raised and closed to each other (Figs 1A, E, G, 2A–D, 3A). Antennae slender, distinctly longer than body, scape gradually thickened from middle to apex, apex of scape nearly extending to base of pronotum, scape longer than antennomere III, antennomere III longer than antennomere IV (Figs 1A, E, 2A–D, 3A). Pronotum sub-cylindrical, slightly expanded posteriorly and distinctly longer than wide (Figs 1A, E, 2A–D, 3A). Procoxal cavities closed posteriorly and triangular externally. Mesocoxal cavities opened externally to mesepimera. Scutellum linguiform (Figs 1A, E, 2A–D, 3A). Elytra slender and elongate, gradually constricted from base to middle, and gradually expanded from middle to about apical 1/5, finally gradually constricted towards apex, emarginated apically (Figs 1A, E, 2A–D, 3A). Femora robust, metafemora distinctly shorter than abdominal ventrite I, hind legs distinctly shorter than abdominal ventrite I–III combined (Figs 1B, C, 1F, 3B, C).

#### Diagnosis.

*Ectinogramma* resembles *Elongatopothyne*Breuning (1963) by (1) slender and elongated body form; (2) slender antennae, distinctly longer than body; (3) elongated pronotum; (4) linguiform and densely clothed scutellum; (5) elytra apically emarginated and coarsely and densely punctate; (6) mesocoxal cavites opened externally to mesepimera; and (7) hind legs distinctly shorter than abdomen. *Ectinogramma* can be differentiated from *Elongatopothyne* by the following characters: (1) shorter antennae, less than twice length of body (in *Elongatopothyne* with antennae three times length of body); (2) scape longer than antennomere III (*El.* with scape shorter than antennomere III); (3) antennomere III longer than IV (*El.* with III shorter than IV); and (4) pronotum slightly expanded posteriorly (*El.* with pronotum not expanded posteriorly).

#### Distribution.

Indonesia, Malaysia.

### ﻿ Ectinogramma isosceloides

Taxon classificationAnimaliaColeopteraCerambycidae

Thomson, 1864

BF84F69C-D50D-5316-9AB7-1C4AA03F92A9

Figs 1A–H , 2A–D

Ectinogramma Isosceloïdes Thomson, 1864: 96 (type locality: “Malasia”).
Ectinogramma isosceloides Gemminger and Harold 1873: 3131 (catalogue); Aurivillius 1923: 359 (catalogue); Breuning 1961: 202 (catalogue).
Ectinogramma collare Pascoe, 1866: 266 (type locality: “Penang, Presqu’île de Malacca, Malasia”), pl. XXVIII, fig. 10; Gemminger and Harold 1873: 3131 (catalogue); Aurivillius 1923: 359 (catalogue).
Spinogramma ruficollis Breuning, 1959: 8 (type species: “Kuala Lumpur, péninsule de Malacce, Malaysia”); Breuning 1961: 202 (catalogue); Cools 1993: 44. syn. nov.

#### Note.

Comparative analyses of the holotype of *Ectinogrammaisosceloides* Thomson (Fig. 1A–C) and *Spinogrammaruficollis* Breuning (Fig. 1E–G) reveal that the latter species is synonymous with *E.isosceloides*.

#### Body length and humeral width of elytra.

Male: 23–25 mm, 2.8–5 mm (five individuals). Female: 20.5 mm–27.5 mm, 2.8–5 mm (eleven individuals).

#### Type material examined.

*Ectinogrammaisosceloides* Thomson, 1864: **holotype**, ♀ (MNHN), *Th.* TYPE (printed with black ink on a rectangular white label, with a transversal black line between “*Th.*” and “TYPE”, with a black inside border) / Ex Musæo JAMES THOMSON (printed with black ink on a rectangular white label, with a black inside border)/ MUSéUM PARIS 1952 coll. R. OBERTHÜR (printed with black ink on a rectangular white label)/ *Isosceloïdes Thoms. Type Malas* (handwritten with black ink on a rectangular yellow label, the label with a wide yellow outside border and a narrow yellow inside border, with three transversal yellow lines in the inside border) / HOLOTYPE (printed with black ink on a rectangular red label) / HOLOTYPE *Ectinogrammaisosceloides* Thomson, 1864 (printed with black ink on a rectangular white label) / MNHN, Paris EC30552 plus a QR (quick response) code (printed with black ink on a rectangular white label); examined from four photographs (Fig. 1A–D).

*Spinogrammaruficollis* Breuning, 1959: **holotype**, ♀ (RBINS), *Coll. I. R. Sc. N. B.* Malaisie: (printed with black ink on a rectangular yellow label, with a transversal black line between “*Coll. I. R. Sc. N. B.*” and “Malaisie:”) / Malacce Kuala-Lumpur Stankovič (handwritten with black ink on a rectangular white label) / *Spinogrammaruficollis* mihi Typ Breuning det. (“*Spinogrammaruficollis* mihi Typ” handwritten and “Breuning det.” printed with black ink on a rectangular white label) / Cf.: Bull.Inst.r.Sci.nat. Belg.1959,35,6:8 (handwritten with black ink on a rectangular white label) / Holotype (Printed with black ink on a rectangular red label with a black inside border); examined from four photographs (Fig. 1E–H).

#### Additional material examined.

**Malaysia**: • 1♂ (CWW), Mt. Trus-Madi, Sabah, Borneo, 15.IV.2005, leg. local collector, examined from one photograph (Fig. 2A); • 1♂ (CWW), Mt. Trus-Madi, Sabah, Borneo, 14.IV.2007, Cope collection, examined from one photograph; • 1♀ (LPSNU), Mt. Trus-Madi, Sabah, Borneo, 27.VI.2022, leg. Dong-Shuo Liu (Fig. 2B); • 1♀ (CWW), Crocker Range, Sabah, Borneo, 19.V.2009, Cope collection, examined from one photograph; • 1♀ (CDJH), Mt. Trus-Madi, Sabah, Borneo, alt. 2000 m, III/V.1998, leg. local collector, examined from one photograph; • 1♀ (CDJH), Crocker Range, Sabah, Borneo, IV.1998, leg. local collector, examined from one photograph; • 1♀ (CDJH), Crocker Range, Sabah, Borneo, 10.III.1999, leg. local collector, examined from one photograph; • 1♂ (CDJH), Crocker Range, Sabah, Borneo, alt. 1000 m, 18.III.2011, leg. local collector, examined from one photograph. **Indonesia**: • 1♂ (CSG), Mt. Bawang, Kalimantan, Borneo, alt. 245 m, V.2019, leg. local collector, examined from one photograph (Fig. 2C); • 1♀ (CST), Madi vill. env., Mt. Bawang, Singkawang region, SW Kalimantan, Kalimantan Barat pr., alt. 1000–1500 m, VI.2018, leg. local collector, examined from one photograph (Fig. 2D); • 1♀ (CST) Mt. Bawang, W. Kalimantan, III.2016, leg. local collector; • 1♂1♀ (CDJH), Mt. Bawang, West Kalimantan, VI.2016, leg. local collector, examined from two photographs; • 1♀ (CDJH), Mt. Bawang, West Kalimantan, IV.2016, leg. local collector, examined from a single photograph.

#### Distribution.

Malaysia (Kuala Lumpur, Penang, Sabah), Indonesia (Kalimantan).

#### Remarks.

After examining some males and females, we found that the male (Fig. 2A, C) is very similar to the female in the body shape, color, pubescence, but they can be distinguished from females by the pedicel with an apical spinous projection, which is longer than the pedicle (Fig. 2A, C).

The genders of the holotype of *Ectinogrammaisosceloides* (Fig. 1A–C), *Ectinogrammacollare* (pl. XXVIII, fig. 10 in Pascoe 1866) and *Spinogrammaruficollis* (i.e., *E.isosceloides*; Fig. 1E–G) were not provided in the original literature; however, based on our examination, the three holotypes are females because the pedicel lacks the spiniform projection found in males.

### ﻿ Ectinogramma ochreovittata

Taxon classificationAnimaliaColeopteraCerambycidae

(Breuning, 1947) comb. nov.

44D07D11-F761-5E3E-BF7F-52483930BC86

Fig. 3A–D

Spinogramma ochreovittata Breuning, 1947: 62 (type locality: “Sumatra, Indonesia”); Breuning 1961: 202 (catalogue).

#### Remarks.

Breuning (1959) mentioned the differences between the two species of *Spinogramma* (i.e., *S.ruficollis* and *S.ochreovittata*) in his description of *S.ruficollis*; however, there are several key characters that we would like to add that were not mentioned by Breuning. As stated above, *S.ruficollis* is synonymous with *E.isosceloides*; therefore, this species is compared with *E.ochreovittata*, and the found differences are detailed below.

The redescription of *Ectinogramma* reveals many common characteristics between the two species, *E.ochreovittata* and *E.isosceloides*. *Ectinogrammaochreovittata* can be distinguished from the later species by the following characteristics: (1) scape, pedicel and antennomere III dark reddish; basal half and apex of antennomere IV dark reddish, rest of surface reddish brown; antennomeres V–VIII reddish brown except apex reddish brown; antennomere IX reddish brown (*E.isosceloides* with entire antennae black); (2) pedicel with a trochiformis and apically outward pointed projection, this projection shorter than the pedicel (*E.isosceloides* the pedicel with a spinous and apically outward pointed process in males, this process is longer than the pedicel; pedicle lacking a spinous and apically outward pointed process in females); (3) prothorax black (reddish brown in *E.isosceloides*); (4) pronotum with mid-dorsal yellowish-brown pubescence vitta on disc and sides with yellowish-brown pubescence vittae on the anterior ^2^/_3_ and sparsely scattered short white pubescence behind (pronotum of *E.isosceloides* sparsely covered with short yellowish-brown pubescence); (5) pronotal disc with transverse wrinkles (disc of *E.isosceloides* without transverse wrinkles); (6) scutellum densely clothed with short yellowish-brown pubescence (scutellum of *E.isosceloides* densely clothed with short yellowish pubescence); (7) elytra with a yellowish-brown pubescence band along suture, and side with reddish-brown area on anterior ^2^/_5_ which gradually constricts apically, the reddish-brown area covered with sparse yellowish-brown pubescence (elytra of *E.isosceloides* with a pale yellowish pubescence band along suture, and side with reddish-brown area anteriorly, the reddish-brown area covered with short yellowish-brown pubescence); (8) mesanepisternum, mesepimeron and metanepisternum sparsely clothed with short white pubescence (*E.isosceloides* with mesanepisternum, mesepimeron and metanepisternum densely clothed with short yellowish pubescence); and (9) tibiae and femora of middle legs and tibiae of hind legs reddish brown (*E.isosceloides* with tibiae and femora of middle legs and tibiae of hind legs black).

#### Type material examined.

***Holotype***, gender unknown (NHRS), Sumatra, A. Koller (printed with black ink on a rectangular white label) / *Spinogrammaochreovittata* mihi Typ Breuning det. (“*Spinogrammaochreovittata* mihi Typ” handwritten and “Breuning det.” printed with black ink on a rectangular white label) / NHRS-ALJB 000000775 (printed with black ink on a rectangular white label) / Typus (printed with black ink on a rectangular red label with a black inside border) / 3808E93 + (printed with black ink on a rectangular blue label); examined from four photographs (Fig. 3A–D).

#### Distribution.

Indonesia (Sumatra).

## Supplementary Material

XML Treatment for Ectinogramma

XML Treatment for Ectinogramma isosceloides

XML Treatment for Ectinogramma ochreovittata

## ﻿Additional information

### Use of AI

No use of AI was reported.

### Author ORCIDs

Gui-Qiang Huang https://orcid.org/0000-0002-0063-8157

Yao-Lan Li https://orcid.org/0009-0009-6222-5997

Hai-Jun Lu https://orcid.org/0009-0007-1561-1030

Dong-Shuo Liu https://orcid.org/0000-0002-9996-842X

### Data availability

All of the data that support the findings of this study are available in the main text.
